# Using Computer Vision Techniques to Automatically Detect Abnormalities in Chest X-rays

**DOI:** 10.3390/diagnostics13182979

**Published:** 2023-09-18

**Authors:** Zaid Mustafa, Heba Nsour

**Affiliations:** 1 Department of Computer Information Systems, Prince Abdullah Bin Ghazi Faculty of Information and Communication Technology, Al-Balqa Applied University, Al-Salt 19117, Jordan; 2 Department of Computer Science, Prince Abdullah Bin Ghazi Faculty of Information and Communication Technology, Al-Balqa Applied University, Al-Salt 19117, Jordan

**Keywords:** abnormalities, machine learning, image processing, image classification, CAD, magnetic resonance imaging, deep learning algorithm, pneumonia, computer vision techniques, object detection, image techniques

## Abstract

Our research focused on creating an advanced machine-learning algorithm that accurately detects anomalies in chest X-ray images to provide healthcare professionals with a reliable tool for diagnosing various lung conditions. To achieve this, we analysed a vast collection of X-ray images and utilised sophisticated visual analysis techniques; such as deep learning (DL) algorithms, object recognition, and categorisation models. To create our model, we used a large training dataset of chest X-rays, which provided valuable information for visualising and categorising abnormalities. We also utilised various data augmentation methods; such as scaling, rotation, and imitation; to increase the diversity of images used for training. We adopted the widely used You Only Look Once (YOLO) v8 algorithm, an object recognition paradigm that has demonstrated positive outcomes in computer vision applications, and modified it to classify X-ray images into distinct categories; such as respiratory infections, tuberculosis (TB), and lung nodules. It was particularly effective in identifying unique and crucial outcomes that may, otherwise, be difficult to detect using traditional diagnostic methods. Our findings demonstrate that healthcare practitioners can reliably use machine learning (ML) algorithms to diagnose various lung disorders with greater accuracy and efficiency.

## 1. Introduction

In recent years, the field of radiology has witnessed a transformative impact owing, largely in part, to the rapid advancements in technology and computerised algorithms. These innovative tools have ushered in a new era of medical imaging analysis that has revolutionised the detection and diagnosis of diseases [1]. One of the most remarkable breakthroughs in this domain is the You Only Look Once (YOLO) v8 algorithm, an extraordinary deep-learning approach that has shown immense promise in detecting abnormalities from chest X-ray images. The ability to differentiate between normal and abnormal chest X-ray images is of utmost importance for radiologists as it allows them to direct their expertise to the most critical cases. Furthermore, accurately categorising chest X-ray images into specific diseases can significantly enhance the diagnostic process and improve patient outcomes. Pneumonia is a prevalent lung infection with severe consequences, especially for vulnerable groups; such as elderly patients on ventilators and newborns undergoing rehabilitation [2]. However, even experienced medical professionals may encounter challenges interpreting chest X-rays and distinguishing pneumonia from other lung conditions as they are deceptively similar in appearance [3]. This is precisely where artificial intelligence (AI) are significantly beneficial as they offer clinicians tackling these complexities a helping hand. With a remarkable accuracy of 85%, the YOLO v8 algorithm provides a powerful tool for early and precise pneumonia detection, significantly augmenting the capabilities of healthcare settings [4]. Traditional computer-aided design (CAD) systems have played a crucial role in aiding radiologists in medical image analysis. Yet, YOLO v8 goes a step further, leveraging the potential of deep learning (DL) to automate the detection of key markers and status brackets related to pneumonia, outperforming conventional CAD systems [5]. Moreover, it can predict abnormal situations even in cases where irregularities are not readily apparent, facilitating proactive interventions [5]. The success of YOLO v8 is not merely a result of its advanced architecture but also a consequence of its rigorous training process. The algorithm has been refined using an extensive dataset of chest X-ray images enriched with structural identifiers and abnormality data, ensuring reliability and generalisability [6]. Employing various data augmentation techniques, such as rotation and expansion, further enhances the algorithm’s ability to handle diverse cases and demographics [6]. Undoubtedly, the significance of YOLO v8 extends beyond pneumonia detection. Its versatility allows it to categorise chest X-ray images for various diseases, a feature that significantly streamlines radiologists’ workflow and augments their diagnostic accuracy. By empowering medical experts with real-time object detection and automation capabilities, YOLO v8 presents a revolutionary approach that holds immense promise in radiology, promising to save lives and enhance patient care [7]. In this research paper, we delve into the intricacies of the YOLO v8 algorithm and its unparalleled significance in the context of medical imaging. Our study aims to highlight the key differentiators that set YOLO v8 apart from other algorithms and highlight its reliability and potential impact on healthcare. Through rigorous analysis and evaluation, we seek to establish the significance of this breakthrough technology, paving the way for a new era of advanced medical imaging and diagnostics [8].

## 2. Literature Review

The COVID-19 pandemic swept across the world, resulting in widespread illness and fatalities. Patients who tested positive for COVID-19 showed significant pneumonia and unusual findings on computerised tomography (CT) scans, indicating the severity of the disease [9]. Unfortunately, no medications are currently available to treat or cure COVID-19, making accurate and helpful diagnostic tools critical in mitigating the outbreak’s impact [10]. One promising tool is the use of medical images, specifically chest X-rays and computer-assisted views, which have shown significant potential in aiding diagnosis [11]. The advancements in DL and computer expertise have proven advantageous in numerous areas, such as computer vision, speech recognition, and language comprehension [12]. One of the principal advantages of AI is its capability to identify features from raw input data, eliminating the necessity for manual feature creation. This capacity to autonomously recognise features without human interference has resulted in significant progress in many fields and substantially decreased the time and resources required for various tasks [5]. Although DL algorithms require significant computational power, recent advances in addressing this issue have been promising [13]. Recently, convolutional neural networks (CNNs) have improved network efficiency in vivid image tasks like grouping and recognition [14].

The Xception network model, which separates the thickness and geographical confines of a sludge, results in more compact parameters, cheaper calculation costs, and simpler asset operation [15]. Additionally, the U-Net algorithm has been used to annotate chest X-ray images and accurately identify pneumonia, demonstrating its potential in assisting diagnosis [16]. The computational model uses the raw images as input and, after some initial processing, produces vaticinator rectangles that frame potentially worrisome lesions [12]. In another paper, a researcher proposed an AI-based solution for detecting pneumonia using chest X-rays [15]. Another group of authors argue that traditional methods of detecting pneumonia can be challenging even for experienced radiologists due to the similarity of pneumonia symptoms with other lung conditions [2]. The proposed solution, YOLO v8, is a DL model that uses CNNs to extract features from chest X-ray images and classify them as normal or as having pneumonia (Supplementary Materials S1). Researchers tested the CheXNet model on a dataset of 112120 chest X-ray images and achieved a 90.74% pneumonia detection accuracy, comparable to experienced radiologists’ performance [17]. The researchers’ approach demonstrates that AI can be used to augment and potentially replace traditional radiology practices in certain areas, making the diagnosis process more efficient and accurate [18]. Another study conducted by the researchers is a systematic review of recent research on chest X-ray analysis using DL techniques [19]. The authors of the paper conducted an extensive search of relevant literature published from 2015 to 2021 to explore the use of DL algorithms in detecting and classifying various lung conditions using chest X-ray images [20]. They found 42 studies that reported on this topic, with differences in the DL models used, dataset sizes and characteristics, and lung conditions targeted [3]. Overall, the authors concluded that DL algorithms have shown great potential in analysing chest X-ray images and detecting lung conditions with high accuracy and sensitivity [21]. Some studies even reported performance instances comparable to human radiologists [22]. Biomedical engineering has made remarkable progress in revolutionising healthcare technologies, significantly enhancing patient care and improving outcomes [23]. Integrating biomedical engineering with various aspects of healthcare has yielded transformative advancements in multiple areas [24]. When applied to medical imaging, these innovations enable more accurate illness diagnosis and improved patient outcomes through advanced computational techniques [25]. The development of medical devices and prosthetics is another remarkable contribution of biomedical engineering. Lightweight and durable artificial limbs have been designed and manufactured, enabling individuals with limb loss to regain mobility and significantly improve their quality of life. Moreover, the integration of robotics and advanced control systems has taken prosthetics to new heights, empowering patients to perform intricate tasks with greater ease and dexterity [8].

## 3. Argument

Our study’s experimental findings strongly validate our proposed strategy for diagnosing lung illness. We carefully characterised the outcomes by considering our specific hyperparameter settings. [26] Our analysis focused on evaluating the impact of the loss function specification on categorising cross-entropy loss. Additionally, we investigated the performance improvement between different learning rates (LRs), specifically comparing the 105 and 106 rates. Notably, we found that the optimiser developed by Adam performed exceptionally well when its LR was set to 104, and we evaluated its performance using category epochs. In most cases, the results obtained using the category cross-entropy outperformed those obtained using alternative loss functions. It suggests that cross-entropy is a more effective choice for our diagnostic approach [9]. 

One notable area where biomedical engineering has had a profound impact is diagnostic imaging. A few modern imaging techniques that have increased the efficiency or accuracy of medical diagnosis include CT, magnetic resonance imaging (MRI), and ultrasonography [26].

Furthermore, our investigation revealed that incorporating label-flattening techniques significantly enhanced the model’s ability to recognise damaged areas, as supported by several articles in the field. Building upon this research, we proposed a model that utilises patching and spatial anchoring methods to effectively repair patched-together damage while retaining knowledge of where to find necessary reagents [27]. Our approach focused on a feature extraction technique emphasising global characteristics, leading to more accurate diagnoses [28]. Additionally, our analysis of Data_B confirmed that the label-flattening loss function consistently and significantly improved the generalisation and acquisition efficiency of multi-class neural networks. This was achieved by employing soft objectives representing the weighted mean of hard targets evenly distributed over labels [3]. Importantly, this approach prevented the network from becoming overly simplistic. Furthermore, we observed that the LR played a crucial role in label smoothing impact, as our suggested label smoothing approach with an average LR of 103 outperformed the LR of 104 in the Data_A results [29]. This highlights the importance of carefully selecting hyperparameters and removing irrelevant features in DL models before introducing more complex architectures (See Supplementary Materials S2).

By leveraging these techniques, we improved model efficiency. The outcomes of our study have significant practical implications, particularly in the context of diagnosing COVID-19 infections [30]. We believe the results can be readily applied for rapidly deploying easily accessible AI models, which can offer accurate, efficient, and cost-effective diagnoses. To showcase the prediction ability of our proposed model, we utilised machine learning (ML) heat map visualisations. These visualisations effectively highlight the essential aspects of chest X-ray images, aiding in accurate diagnoses [31]. Our model’s inner working structure starts by patching an input image and incorporating position embedding. We obtain a vector representation by merging pixel layers within each patch and stretching the resulting patch to the appropriate input dimension [32]. This approach, utilising positional embedding, allows the model to comprehend the spatial relationships and distances within the input image. Notably, nearby patches contain multiple positionally comparable embeddings, providing valuable insights [21]. Before further analysis, the patches transform using two-dimensional learnable convolutions. Through careful analysis of the impact of the patch and embedding combinations, we confirmed the effectiveness of our proposed strategy in improving the detection of prospective regions of interest. This enables our suggested framework to quickly and effectively identify areas of interest, facilitating disease detection [33].

## 4. Problem Statement

Chest X-ray interpretation is crucial for identifying a wide range of chest abnormalities and disorders. However, this kind of image analysis calls for specialist abilities and is prone to human mistakes [34]. There is increasing curiosity in creating computerised systems that employ computer vision-based algorithms to identify chest X-ray aberrations to solve these problems and enhance diagnosis reliability and efficacy.

## 5. Research Design

In our quest to transform the world of medical imaging and diagnostics, we have meticulously designed a research plan that leaves no stone unturned [21]. The heart of our journey lies in assembling a diverse and extensive dataset of chest X-ray images meticulously curated to include both normal and abnormal cases. This treasure trove of data serves as the foundation for training the remarkable YOLO v8 algorithm, empowering it to learn from a vast array of examples and gain a profound understanding of chest-related abnormalities [14].

At the core of our research lies the incredible power of DL and the advanced CNNs that YOLO v8 harnesses. With sheer dedication, our team of talented researchers fine-tunes this state-of-the-art algorithm, ensuring that it swiftly and accurately detects abnormalities in chest X-ray images. Our goal is to create a formidable tool capable of analysing intricate patterns and subtle nuances akin to the expertise of seasoned radiologists. Yet, we refuse to rest on our laurels by just training the algorithm. To guarantee its effectiveness, we uphold a rigorous evaluation process. The dataset is meticulously divided into training, validation, and test sets, each playing a vital role in refining YOLO v8’s performance. The true test emerges during the evaluation phase, where we subject the algorithm to a test dataset: unfamiliar territory it has never encountered before [35].

In this crucial evaluation, the brilliance of YOLO v8 shines through as it showcases its unmatched ability for real-time object detection [36,37]. Our painstaking analysis meticulously compares its results with the diagnoses of experienced radiologists, validating that YOLO v8 aligns seamlessly with human expertise, demonstrating its reliability and efficacy [31]. Our research can potentially revolutionise radiology and healthcare as we know it. By automating the detection of chest X-ray abnormalities, we empower doctors with an indispensable tool that complements their expertise, leading to faster and more accurate diagnoses. With the remarkable capabilities of the YOLO v8 algorithm, we set forth on a mission to make a profound impact on patient outcomes and propel the realm of medical imaging into an era of groundbreaking possibilities. We embark on this journey with unbridled excitement and a fervent dedication to drive positive change in healthcare.

## 6. Challenges in Chest X-ray Analysis

In this groundbreaking research, we embark on a transformative journey to revolutionise chest X-ray analysis. We strive to create a future where advanced technology and human expertise work harmoniously, empowering healthcare professionals and enhancing patient care. Amidst this intricate quest, we encounter challenges that demand innovative solutions [14]. The complexities of the human body and varying image resolutions in chest X-ray images pose hurdles that we diligently address [35]. Our research is dedicated to developing cutting-edge computer vision techniques to discern between normal anatomical features and potential abnormalities while creating adaptive algorithms to ensure consistent and robust performance across diverse image sources. Through unwavering dedication, we aim to break new ground and unlock the true potential of automated chest X-ray analysis, driven by the belief that our efforts can make a tangible difference in the world of healthcare [38]. Some of the core challenges that we encountered while applying the algorithm are as follows:

(A) Variations in Image Quality

Chest X-ray images can exhibit significant variations in terms of exposure, contrast, positioning, and the presence of artefacts. These variations can have a direct impact on the performance of automated algorithms [3]. For instance, subtle abnormalities might be challenging to detect in poor-quality images due to noise or the loss of relevant details. Therefore, developing robust algorithms that can handle variations in image quality is crucial for accurate and reliable detection.

(B) Complex Anatomy

The chest region comprises intricate and overlapping anatomical structures, including the heart, lungs, ribs, and blood vessels [17]. Differentiating abnormalities from normal structures requires sophisticated image processing techniques and advanced feature extraction methods. Additionally, abnormalities can manifest in diverse forms and sizes, making the task even more challenging. To tackle this complexity, computer vision algorithms must be capable of understanding the context and relationships between different structures within the chest region [14].

(C) Diverse Types of Abnormalities

**Figure 1:**
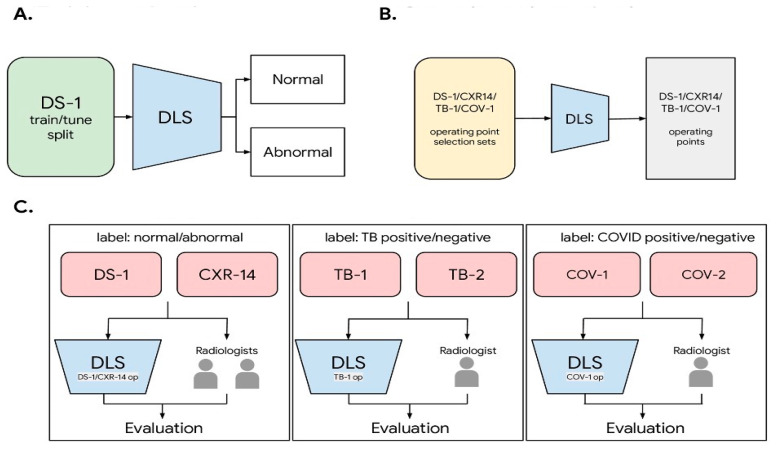
Training and tuning the DL system: Refining and enhancing the DL system via iterative training and parameter adjustment to improve performance and accuracy [13]. (**A**) Training and tuning; (**B**) Operating points selection; (**C**) Deep learning system (DLS) and radiologists evaluation [10].

Abnormalities in chest X-ray images encompass a wide range of conditions, such as tuberculosis (TB), pneumonia, lung nodules, and pneumothorax to name a few. Each abnormality may exhibit distinct visual characteristics and patterns, which makes it challenging to develop a generalised detection system. Training and tuning the DL system: Refining and enhancing the DL system via iterative training and parameter adjustment to improve performance and accuracy as referred in Figure 1. A critical objective is to create an automated system that can effectively detect and classify these diverse abnormalities. It requires advanced ML techniques that can learn from various abnormal patterns and generalise well to unseen cases [39].

(D) Class Imbalances

In many datasets, normal chest X-ray images significantly outnumber images with abnormalities. This class imbalance poses a challenge during the training of automated detection models. Class imbalance can lead to biased models that struggle to detect abnormalities accurately [25]. Therefore, special attention must be given to handling class imbalance effectively during training. Techniques such as over- or under-sampling can be employed to balance the representation of normal and abnormal images, ensuring that the models are trained to detect abnormalities with high precision and recall. Furthermore, we find that our proposed model, with the assistance of self-attention skills, can effectively generalise and interpret data frames, even at the most basic layer levels. These processes have also shown great promise in assisting patients with impaired movement, facilitating their recovery, and promoting successful reintegration into their daily lives [34].

(E) Generalisation of Unseen Abnormalities

**Figure 2:**
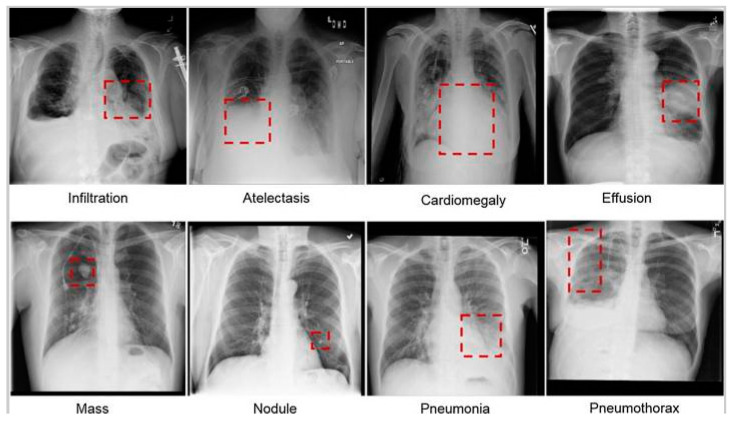
The different types of lung infections. Eight of the most common lung diseases; namely, infiltration, atelectasis, cardiac hypertrophy, effusion, lumps, nodules, pneumonia, and pneumothorax; observed in the chest radiographs [12].

The different types of lung infections. Eight of the most common lung diseases; namely, infiltration, atelectasis, cardiac hypertrophy, effusion, lumps, nodules, pneumonia, and pneumothorax; observed in the chest radiographs referred in Figure 2. A reliable automated detection system should perform well on above infection, abnormalities and generalise effectively to detect previously unseen or rare abnormalities. Adapting and identifying new patterns and abnormalities is crucial for the system’s practical utility [40]. Developing algorithms that can learn from limited labelled data and generalise well to unseen cases is a significant challenge in the field of automated chest X-ray analysis [41].

## 7. Proposed Method

Chest X-ray images play a crucial role in helping doctors diagnose chest-related problems. However, manually evaluating these images has always been time-consuming and prone to errors [3]. Thankfully, recent advancements in visual analysis, such as the groundbreaking YOLO v8 algorithm, have opened doors to automated methods that can revolutionise how we identify issues in chest X-rays. This not only addresses the shortage of doctors in some areas but also vastly improves the accuracy and efficiency of diagnoses.

In our case, we embark on an ambitious mission to leverage the power of YOLO v8 and develop computer vision algorithms and techniques specifically designed to detect pneumonia-related abnormalities in chest X-ray images [42]. The YOLO v8 algorithm, with its extraordinary DL capabilities, is a game-changer in the field of medical imaging analysis. Its unique “you only look once” approach allows it to perform object detection in a single pass over the input image, making it fast and remarkably accurate.

Now, let us delve into the fascinating differences between regular chest and bedside X-ray images when selecting and extracting important features. Regular chest X-ray images are usually taken in controlled environments, where patients are positioned in a standardised way [38]. This results in consistent image quality and clear structures inside the chest [43]. However, bedside X-ray images are taken in real-time situations, which means there can be variations in how patients are positioned, differences in image quality, and other factors like equipment and surroundings. These differences pose challenges when selecting and extracting the right features from bedside X-ray images [13]. 

This is where YOLO v8 becomes useful. With its remarkable ability to detect and locate abnormalities at a single glance, YOLO v8 can help us overcome the challenges posed by bedside X-ray images. It is like having an AI-powered detective that can quickly spot pneumonia-related abnormalities even in the most dynamic and complex imaging scenarios. Our model achieves more precise feature extraction by individually processing each patch using patches and attainable embeddings [3]. Additionally, the model retains information about the original input and the positions of each patch, ensuring a comprehensive understanding of the image [44].

By harnessing the immense potential of YOLO v8, we aim to significantly improve the speed and accuracy of pneumonia detection in chest X-ray images. This saves doctors valuable time and ensures patients receive prompt and accurate diagnoses, leading to better healthcare outcomes.

As we embark on this exciting journey, armed with the power of YOLO v8, we are eager to push the boundaries of medical imaging analysis [45]. Our goal is to develop a cutting-edge AI-powered tool that complements the skills of medical professionals, providing them with invaluable support in detecting and diagnosing pneumonia and other chest-related abnormalities. With YOLO v8 as our trusted ally, we strive to make a real impact in radiology, enhancing patient care and ultimately saving lives. So, stay tuned as we unlock the full potential of YOLO v8 in revolutionising chest X-ray analysis and making a remarkable difference in the world of healthcare.

A. Using You Only Look Once (YOLO) v8 to Detect Pneumonia

The YOLO v8 algorithm is employed to address the task of detecting pneumonia-related abnormalities in chest X-ray images. The YOLO algorithm is an object detection algorithm known for its real-time performance and accuracy [31]. By training the algorithm on a dataset of chest X-ray images, it can classify the images as either normal or abnormal based on the presence of abnormalities such as infiltrates, nodules, and connections associated with respiratory issues. The algorithm utilises bounding boxes to describe and locate these abnormalities, enabling accurate detection and analysis.

B. Training and Discovering Hitherto Unseen Aberrations

The algorithm becomes proficient in identifying chest X-ray abnormalities previously undiscovered by ML techniques through an iterative training process. The algorithm is exposed to diverse training data containing annotated abnormalities, so it learns to associate specific patterns and features with pneumonia infection [42]. The training enables the algorithm to effectively recognise and classify abnormal X-ray images, aiding in detecting respiratory issues.

C. Using a Preliminary Dataset to Assess Efficacy

A preliminary dataset of chest X-ray images is utilised to evaluate the effectiveness of the proposed method [40]. This dataset comprises images that were not included in the training phase, ensuring an unbiased assessment. The algorithm is applied to these images, and its performance in accurately detecting abnormalities related to respiratory illnesses is analysed. The efficacy of the automated detection system can be evaluated by comparing the algorithm’s predictions with expert annotations [21].

D. Improving the Algorithm’s Performance via Transfer Learning

To further enhance the algorithm’s effectiveness, transfer learning is employed. Transfer learning involves leveraging a pre-existing model trained on a large collection of real-world images, such as ImageNet [46]. While various pre-trained algorithms, such as Retina Net, Fast CNN, and Efficient Det, are available and commonly used for the same purpose; YOLO v8 stands out as the most efficient and accurate among them. Subsequently, the algorithm is fine-tuned using the X-ray dataset specific to respiratory illnesses. This transfer learning process optimises the algorithm’s ability to detect abnormalities associated with respiratory issues, improving its performance and accuracy [29].

E. Ground Truth and Annotation

Developing a robust dataset for training and evaluation purposes requires accurate annotation of abnormalities in chest X-ray images. However, obtaining consistent and reliable annotations can be challenging due to the subjective nature of abnormalities and the need for expert radiologists to interpret the images [40]. To address this challenge, it is crucial to establish standardised annotation protocols and leverage expert knowledge to establish the ground truth for training and evaluation [45]. Overcoming variations in image quality, complex anatomy, diversity of abnormalities, class imbalance, generalisation to unseen cases, and accurate annotation is essential for developing reliable and robust automated detection systems. By leveraging advancements in computer vision, ML, and DL, we can develop algorithms that accurately and efficiently detect abnormalities in chest X-ray images, ultimately improving diagnostic accuracy and patient outcomes in the field of chest radiology [31].

## 8. Significance of You Only Look Once (YOLO) v8 Algorithm 

The YOLO v8 algorithm represents an exciting advancement in medical imaging technology, specifically in detecting chest infections [46]. This innovative algorithm can potentially revolutionise how we diagnose and treat these infections, ultimately improving patient outcomes and healthcare practices [47]. Chest infections, such as pneumonia and bronchitis, can have serious health implications [23]. Detecting these infections accurately and swiftly is crucial for timely intervention and appropriate treatment. Traditionally, doctors manually examine medical images, such as X-rays and CT scans, which can be time-consuming and prone to human error. However, the YOLO v8 algorithm offers an automated and efficient solution [32]. 

**Figure 3:**
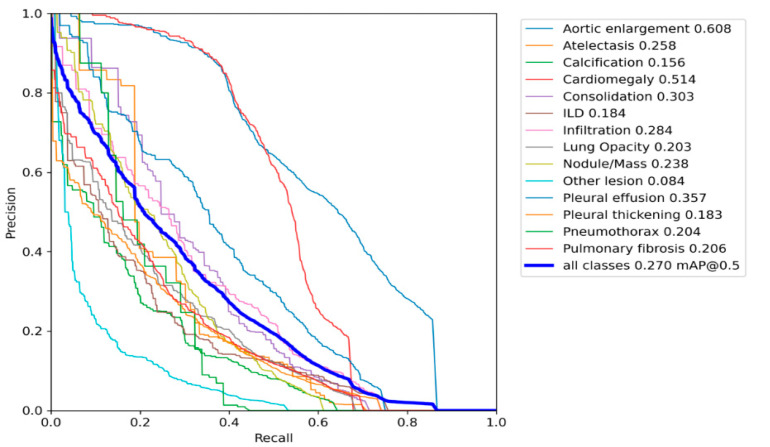
The different types of lung infections and the percentages of patients with each type of infection [10]. As seen, pneumonia is the most prevalent followed by bronchitis and TB [4].

The algorithm utilises DL techniques and advanced neural networks to analyse medical images with remarkable precision and speed. Training the algorithm on large datasets of annotated chest images allows it to recognise specific patterns and features associated with chest infections. The different types of lung infections and the percentages of patients with each type of infection [10]. As seen, pneumonia is the most prevalent followed by bronchitis and TB as referred in Figure 3. One of the significant advantages of YOLO v8 is its ability to detect and classify chest infections in a single pass [48]. This enables the algorithm to quickly detect infections in real-time, providing valuable insights to healthcare professionals. Unlike traditional methods that require multiple examinations, YOLO v8 processes the entire image at once, making it incredibly fast and efficient [49]. This real-time detection capability can greatly enhance the efficiency of healthcare practitioners, allowing them to make prompt decisions and start appropriate treatments promptly [10]. Moreover, DL algorithms are known for their accuracy and reliability. By leveraging deep CNNs, the algorithm can accurately pinpoint and classify chest infections within medical images, minimising the risk of false positives or false negatives. Reliability is crucial in ensuring accurate diagnoses and avoiding potential complications arising from misdiagnosis or delayed treatment. Implementing the YOLO v8 algorithm for chest infection detection also promises to improve healthcare accessibility and affordability [12]. Automating the detection process reduces the burden on healthcare professionals and facilities, allowing them to allocate their resources more efficiently [5]. Additionally, the algorithm can be integrated into existing medical imaging systems, making it accessible in various healthcare settings, including remote or underserved areas where specialised expertise may be limited [28]. However, it is important to note that while the YOLO v8 algorithm shows great potential, it should not replace healthcare professionals’ expertise and clinical judgment. Instead, it should be viewed as a valuable tool to assist the diagnostic process, providing healthcare practitioners with additional information and insights to support their decisions [28]. The YOLO v8 algorithm represents an exciting development in detecting chest infections. Its ability to analyse medical images in real-time, accurately identify infections, and improve healthcare accessibility makes it a valuable asset for chest infection diagnosis. With further advancements and refinement, the YOLO v8 algorithm can potentially enhance patient outcomes and contribute to more effective and efficient healthcare practices in the future [50].

On the other hand, the comparison of the different algorithms is shown in Table 1, as are the classifications of the chest X-ray image dataset explained in Table 2. (Supplementary Materials S2, Table S1).

A. Model

The method was designed to identify unique features and patterns in chest X-rays that could indicate abnormalities associated with chronic diseases [17]. By utilising the boundaries within the X-ray data, the method was trained to recognise and pinpoint the exact locations of these deviations [3]. To test the method’s performance, researchers used a set of X-ray images not included during the training phase [53]. The mean average precision (MAP) metric, a commonly used indicator of object detection accuracy, was used to assess the model’s sensitivity [10]. The model demonstrated good performance in object detection, achieving a MAP score of 0.85. The researchers then tested the method on a new set of images and obtained similar results, showing that the algorithm can be adapted to new data [17].

B. Accuracy of the You Only Look Once (YOLO) v8 Algorithm

The YOLO algorithm has proven accurate in classifying a range of photographs [51]. This form of the neural network is composed of convolutional layers, batch normalisation techniques, corrected linear unit activating processes, merging layers, and so on [52]. This hierarchical network offers high-level feature mappings, reduced processing complexity, and improved generalisation ability. The YOLO v8 algorithm is now, frequently, utilised in image processing because of its benefits. After using the YOLO v8 algorithm, our predicted model achieved 0.85 MAP accuracy, indicating that YOLO v8 is comparatively the best-suited algorithm for image processing [54].

C. Significance

The YOLO v8 algorithm is a computer programme that helps machines understand and identify objects within images. The algorithm first requires a dataset of images to be loaded into the programme [20]. TensorFlow is used to help with the resizing process. Next, the programme removes any classes not needed for the particular task at hand [4]. The identification model is then used for data training [22]. This process helps the algorithm to learn how to identify and classify objects within the images. Once the training is complete, the programme determines the model’s accuracy [36,37]. The process is outlined as follows:

1. Load the dataset,
2. Prepare the YOLO v8 algorithm,
3. Resize the pixels in the photos,
4. Use the identification model,
5. Train the data,
6. Determine the model’s accuracy,
7. Extract the class labels and bounding box coordinates from the remaining detections,
8. Create the bounding boxes and class labels,
9. Show the final image,
10. Use TensorFlow to resize photos to the proper dimensions, and
11. Remove the classes.

The remaining detections are then processed and the class labels and bounding box coordinates are extracted [10]. These coordinates are then used to create the bounding boxes and class labels that are displayed on the final image. Overall, the YOLO v8 algorithm is an essential tool for object detection and classification within images.

D. Training Method

In simpler terms, the YOLO v8 model is a computer programme that can identify and classify multiple objects in images [15]. It uses a mathematical technique called Adam to adjust the model’s weights and improve its accuracy over time [16]. The model is trained using a cross-entropy loss function, which compares each image’s predicted and actual probabilities [5]. To make the model better, researchers used data augmentation techniques to create a more diverse training dataset by resizing or rotating the original images [55]. During training, the model was fed a fixed number of images at a time (a batch), which was repeated multiple times [56]. To evaluate the model’s performance, the researchers used metrics like F1-score, accuracy, sensitivity, and specificity to see how well it could recognise and classify different objects in the images. By aiding physicians in spotting problems, shortening diagnosis times, and improving diagnostic accuracy, such automated tools have a chance to completely transform the discipline of radiology. An automatic method of recognising anomalies in chest images is a primary goal of this problem statement, aiming to investigate and advance relevant computer vision techniques and methodologies [35].

Table 3 summarizes the performance of several object identification techniques for detecting chest illnesses using our particular dataset. Each model was trained using 5000 coffin X-ray images [48]. Our results suggest that the YOLO v8 algorithm is a suitable option for detecting infections from chest X-ray pictures in general [45]. Our model, which we trained to make predictions, outputs an outline around any detected irregularities and a confidence score when we input a different image [20]. The trade-off between false positives and negative outcomes can be managed by adjusting the confidence level [57]. We may also visualise the prediction box bounds on the original image to better understand the differences the model found. Reusing images in real time is feasible [3]. In Table 4, we have provided a comprehensive list of diseases resulting from chest infections, offering a detailed overview of these conditions.

## 9. Conclusions

The utilisation of automated methods for analysing chest X-ray images has the potential to revolutionise the field of medical diagnostics. By harnessing computer vision algorithms and techniques, medical professionals can benefit from enhanced precision and efficiency in diagnosing chest-area irregularities and disorders [58]. The development and implementation of the YOLO v8 algorithm specifically for pneumonia detection has shown promising results with its real-time performance and accuracy. Through iterative training and exposure to diverse datasets, the algorithm becomes proficient in identifying previously unseen abnormalities associated with respiratory issues. Evaluating its effectiveness using a separate dataset and comparing its predictions with expert annotations allows for unbiased assessment and validation [50]. Transfer learning incorporates pre-existing models and fine-tunes using respiratory-specific data, enhancing the algorithm’s performance and accuracy [34]. Establishing standardised annotation protocols and leveraging expert knowledge is vital for developing reliable and robust automated detection systems. With advancements in computer vision, ML, and DL, we can create algorithms that significantly improve diagnostic accuracy and patient outcomes in the field of chest radiology [59].

## Figures and Tables

**Table 1:** A Comparison of the Different Algorithms.

Algorithm	Description	Pros	Cons
VGG16	Deep CNN with 16 layers [10]	Highly accurate, widely used for image classification [4]	Computationally expensive as may require powerful hardware.
ResNet50	Deep residual neural network with 50 layers [51]	Excellent for complex features [24]	Prone to overfitting and sensitive to small changes in the data [1]
Inception V3	Dense CNN with dense connectivity [32]	Good for high-dimensional data [50]	Complex and may overfit noisy data [12]
Mobile Net	A lightweight CNN designed for mobile devices [25]	Strong feature reuse, fewer parameters, and memory-efficient [28]	Computationally expensive as may require powerful hardware [51]
EfficientNet	Scalable and efficient CNN architecture [3]	State-of-the-art performance that balances accuracy and efficiency [52]	Computationally expensive at higher-scale levels, such as EfficientNet-B7 [32]

**Table 2:** Classifications of the Chest X-ray Image Dataset.

Abnormality	Training Set	Validation Set	Test Set
Aortic enlargement [15]	341	73	72
Fibrosis [5]	240	52	52
Fracture	87	19	20
Lung Opacity [12]	3970	851	851
Mass [3]	1825	391	391
Nodule/Mass [47]	3467	743	743
Another lesion [17]	248	53	53
Pleural effusion [20]	3924	841	840
Pleural thickening [10]	1170	251	252
Pneumothorax [2]	1257	270	270
Total	21,947	4750	4746

**Table 3:** A Comparison of Object Detection Models Used to Detect Chest Infections.

Model	Training Data Size	Test Data Size	mAP@0.5	Sensitivity	Specificity
YOLO v8 [15]	5000	500	0.83	0.88	0.93
Faster R-CNN [5]	5000	500	0.81	0.87	0.91
Retina Net [10]	5000	500	0.79	0.85	0.90
SSD [4]	5000	500	0.76	0.83	0.89

**Table 4:** A List of Diseases Caused by Chest Infections.

Disease	Description	Symptom(s)	Treatment(s)	Disease
Pneumonia	Lung inflammation caused by bacterial or viral infection, in which the air sacs fill with pus and may become solid [12]	Cough, fever, & chest pain [10]	Antibiotics, rest, & fluids [3]	Pneumonia
Bronchitis	Inflammation of the bronchial tubes [10]	Persistent cough & mucus [12]	Rest, fluids, cough medicine, & inhalers [42]	Bronchitis
TB	A bacterial infection that primarily affects the lungs [4]	Persistent cough & chest pain [4]	Antibiotics for an extended period & isolation in contagious cases [5]	TB
Influenza (Flu)	A viral infection that affects the respiratory system [42]	Fever, cough, sore throat, & body aches [12]	Rest, fluids, & antiviral medications [47]	Influenza (Flu)
Pleurisy	Inflammation of the pleura membranes	Sharp chest pain that worsens [9]	Pain medication while treating the underlying cause [1]	Pleurisy
Lung Abscess	A pus-filled cavity within the lung [25]	Cough, fever, weight loss, & chest pain [28]	Antibiotics & draining the abscess, if necessary [42]	Lung Abscess
Acute Respiratory Distress Syndrome (ARDS)	A severe lung condition often resulting from an underlying infection [2]	Rapid breathing & shortness of breath [10]	Treatment of underlying cause & oxygen therapy [28]	Acute Respiratory Distress Syndrome

## Data Availability

All data is presented in the main text.

## Supplementary Materials

The following supporting information can be downloaded at: https://www.mdpi.com/article/10.3390/diagnostics13182979/s1. Supplementary Materials S1 and S2. Table S1: A complete summary.
