# Supplementary material for: Using Computer Vision Techniques to Automatically Detect Abnormalities in Chest X-rays
Source: Diagnostics (Basel). 2023 Sep 18;13(18):2979. doi: 10.3390/diagnostics13182979 (PMC10530162; doi:10.3390/diagnostics13182979)
Supplement: Supplementary file 1 [file diagnostics-13-02979-s001.zip › Supplementary Materials S1 and S2. Table S1.pdf]

Supplementary Materials S1

When it comes to spotting anomalies in chest X-ray images, the YOLO v8 algorithm has proven to perform better than other object detection algorithms; such as faster regions with convolutional neural networks (Faster R-CNNs), RetinaNet, and single shot detectors (SSDs) [5]. A total of 500 testing and 5000 training images were used in a study using four distinct algorithms [3].

The YOLO v8 algorithm outperformed all three algorithms, achieving a mean average perfect graph of 0.83 at a threshold of 0.5. [44] Additionally, with 0.88 and 0.93 perceptivity and specificity scores, YOLO v8 outperformed the other algorithms. This shows that YOLO v8 performs better at identifying anomalies in chest X-ray images and contrasting them with their typical features [48].

Supplementary Materials S2

Table S1. A complete summary.

Abnormality	Training Set	Testing Set
Pneumonia [7]	3981	1019
Atelectasis [12]	4786	1214
Consolidation [10]	3655	931
Total	12,422	3164
